# Anterior cruciate ligament repair with LARS (ligament advanced reinforcement system): a systematic review

**DOI:** 10.1186/1758-2555-2-29

**Published:** 2010-12-07

**Authors:** Zuzana Machotka, Ian Scarborough, Will Duncan, Saravana Kumar, Luke Perraton

**Affiliations:** 1 International Centre for Allied Health Evidence, University of South Australia, North Terrace, Adelaide, South Australia, 5000, Australia; 2 Wakefield Sports Clinic, 270 Wakefield St, Adelaide, South Australia, 5000, Australia; 3 Wakefield Orthopaedic Clinic, 270 Wakefield St, Adelaide, South Australia, 5000, Australia

## Abstract

**Background:**

Injury to the anterior cruciate ligament (ACL) of the knee is common. Following complete rupture of the ACL, insufficient re-vascularization of the ligament prevents it from healing completely, creating a need for reconstruction. A variety of grafts are available for use in ACL reconstruction surgery, including synthetic grafts. Over the last two decades new types of synthetic ligaments have been developed. One of these synthetic ligaments, the Ligament Advanced Reinforcement System (LARS), has recently gained popularity.

The aim of this systematic review was to assess the current best available evidence for the effectiveness of the LARS as a surgical option for symptomatic, anterior cruciate ligament rupture in terms of graft stability, rehabilitation time and return to pre-injury function.

**Method:**

This systematic review included studies using subjects with symptomatic, ACL ruptures undergoing LARS reconstruction. A range of electronic databases were searched in May 2010. The methodological quality of studies was appraised with a modified version of the Law critical appraisal tool. Data relating to study characteristics, surgical times, complication rates, outcomes related to knee stability, quality of life, function, and return to sport as well as details of rehabilitation programs and timeframes were collected.

**Results:**

This review identified four studies of various designs, of a moderate methodological quality. Only one case of knee synovitis was reported. Patient satisfaction with LARS was high. Graft stability outcomes were found to be inconsistent both at post operative and at follow up periods. The time frames of rehabilitation periods were poorly reported and at times omitted. Return to pre-injury function and activity was often discussed but not reported in results.

**Conclusions:**

There is an emerging body of evidence for LARS with comparable complication rates to traditional surgical techniques, and high patient satisfaction scores. However, this systematic review has highlighted several important gaps in the existing literature that require future prospective investigation. The findings of this review were equivocal with regards to other measures such as graft stability and long term functional outcomes. While the importance of rehabilitation following LARS is well recognised, there is limited evidence to guide rehabilitation protocols.

## Background

Injury to the anterior cruciate ligament (ACL) of the knee is common[1]. A recent population-based study reported that 80% of knee ligament surgery involved the ACL[2]. Following complete rupture of the ACL, insufficient re-vascularization of the ligament prevents it from healing completely, creating a need for reconstruction[3]. Anterior cruciate ligament reconstruction aims to reinstate the functional stability of the knee; in turn, preventing further damage to the menisci and reducing the risk of degenerative osteoarthritis[1,4]. The early success of reconstructive surgery has lead to the progression from open extra-articular stabilisation to arthroscopic anatomic grafting[5-7].

A variety of grafts are available for use in ACL reconstruction surgery. Broadly, all grafts can be placed into one of three categories; autologous grafts, allografts, and synthetic ligaments. Currently, autologous grafts such as hamstring and bone-patella tendon grafts are widely used[8]. Autologous grafts provide a strong scaffold for in-growth of collagen fibers, without the risk of graft rejection[9]. However, autologous grafts carry a risk of harvest site morbidity and require prolonged avoidance of activities during revascularization (while the graft itself has a reduced tensile strength) for a period of up to 12 months[10-12]. Allografts are less common and although they eliminate harvest site morbidity they are more prone to graft rejection, potential viral infection risk, slower healing, and higher failure rates[9,11-13].

Synthetic materials were first used in ACL reconstruction in the 1980 s to improve the strength and stability of the graft immediately post operatively, reduce donor site morbidity and eliminate the potential for disease transmission[11,14,15]. The first synthetic ligaments were associated with high rates of failure and reactive synovitis[14,16]. Over the last two decades with advancing technology, new types of synthetic ligaments have been developed. One of these synthetic ligaments, the Ligament Advanced Reinforcement System (LARS), has recently gained popularity with some orthopedic surgeons and in the media[17,18].

The LARS is a non-absorbable synthetic ligament device made of terephthalic polyethylene polyester fibres[16,19]. The ligament is highly cleaned to remove potential machining residues and oils to further encourage soft tissue in-growth and reduce the risk of reactive synovitis[16]. The intra-articular portion, or scaffold, of the ligament consists of multiple parallel fibres twisted at 90 degree angles[16,20]. This design aims to prevent the fibre breakdown that was previously seen in grafts made from woven materials. Additionally, this design is thought to facilitate even tensioning of the graft fibres during knee movement[16]. The scaffold provides a meshwork for the injured ligament to heal and repair[16,21]. One in-vitro laboratory study has demonstrated cellular growth after six months, subsequent to seeding of human fibroblast and osteoblast like cells onto the LARS[16].

Traditional ACL reconstruction techniques require debriding of the torn ACL fibres and synovial lining that normally envelops the ligament, in order to visualise the position for the graft[17,22]. The LARS surgical technique uses an intra operative image intensifier X-ray to position the tunnels for the LARS through the ACL stump and is therefore able to leave the synovial lining and the torn ACL fibres insitu. The proposed advantage of this technique is reduced trauma to the soft tissues of the knee and less surgical time[17]. The ACL stump is anchored to the meshwork of the LARS to support it in an optimum position while healing. Overall, the LARS surgical technique aims to maximise in-growth of the original ACL tissue, thus preserving some vascular and proprioceptive nerve supply.

### Aim

The potential advantages of LARS are immediate graft stability, reduced rehabilitation time and quicker return to pre-injury function. Despite the current popularity of LARS and some promising clinical results, no systematic review has yet evaluated its effectiveness in terms of these advantages. Therefore the aim of this systematic review was to assess the current best available evidence for the effectiveness of the LARS as a surgical option for symptomatic, anterior cruciate ligament rupture in terms of graft stability, rehabilitation time and return to pre-injury function.

## Methods

This systematic review included studies using subjects with symptomatic, ACL ruptures undergoing LARS reconstruction. Studies using other types of synthetic ligaments and studies of posterior cruciate ligament reconstruction were excluded. Studies published in a language other than English or in non-peer reviewed journals were also excluded. No gender or age limits were placed on the search. All types of comparison groups were included (either control, conservative or alternative surgical intervention). Outcomes of interest included knee stability measures, surgical complication rates, quality of life (QOL), function, and return to sport.

### Search Strategy

Searches were performed in May 2010 on the following databases: MEDLINE, CINAHL, PubMed, SPORTSDiscus, Embase, Ovid, PEDro, Cochrane Controlled Trials Register (DARE and CCRCT) and Google Scholar. No date limits were set. The following keywords were used: 'Ligament augmentation and reconstruction system', OR 'Ligament advancement reinforcement system', OR 'LARS', AND knee, OR 'cruciate ligament', 'anterior cruciate'. Truncation symbols were utilized as appropriate across the different databases. References of retrieved studies were reviewed for further potentially relevant studies. Duplicates were removed to create a master list.

### Hierarchy of evidence

As this review addressed an effectiveness question, only research studies from a quantitative research paradigm were included. The National Health and Medical Research Council (NHMRC) hierarchy of evidence was used to determine the level of evidence of studies (Levels I to IV)[23]. An initial search of the evidence revealed limited published, peer reviewed quantitative studies. Consequently, this review considered all experimental, quantitative research designs within the NHMRC hierarchy of evidence. This approach allowed an exploration of the best available evidence on LARS.

### Quality Evaluation

The modified Law critical appraisal tool was used to appraise the methodological quality of studies[24]. The Law critical appraisal tool was chosen because of its generic nature (applicable to all quantitative research designs) and the authors' prior experience in using the tool. The Law critical appraisal tool contains twelve criteria, which require a yes or no answer, each representing key elements of the methodological quality of a study. Each criterion was given a score of one for yes and a zero score for no answers. Additionally for case study design studies, criterion 11(drop outs), was not applicable and therefore was not given a score. Each study was independently critically appraised by three authors [ZM, IS, SK]. Disagreements were resolved through discussion until consensus was achieved. A copy of this tool is provided in Additional Files.

### Data Extraction

Data was extracted from the individual studies by two reviewers [ZM, IS]. Data relating to study characteristics such as study population, comparison groups, and follow up periods was collected to gain an overview of the included studies. To gain an understanding of the benefits associated with LARS, details of surgical times, complication rates and outcomes related to knee stability were collected. Data regarding QOL, function, and return to sport were collected in order to gain an understanding of patient-relevant outcomes post surgery. Finally, details of rehabilitation programs and timeframes were collected in order to potentially provide recommendations for clinical practice.

### Body of evidence Framework

To better interpret and understand the findings of this review, the National Health and Medical Research Council (NHMRC) body of evidence framework was used[25]. The authors have experience in successfully using this framework and operationalizing it to varying bodies of evidence[26]. This framework considers multiple dimensions of evidence for all included studies, and based on this framework, evidence-based recommendations can be drawn. The components of the NHMRC framework are evidence base, consistency, clinical impact, generalisability, and applicability of the research. The applicability component was not used in this review, as this focuses on the applicability of research findings to specific local settings (for example Australia). As this review was targeted at a larger, international audience, the applicability to a one local health care setting was not considered relevant.

## Results

### Search Results

**Figure 1:**
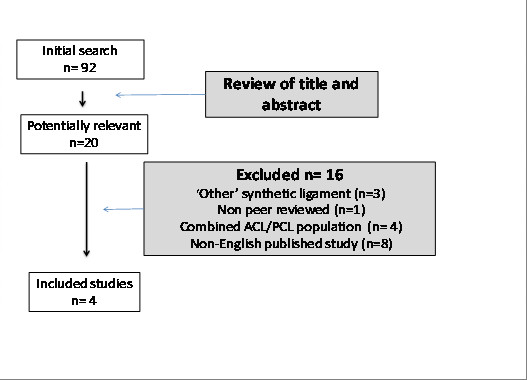
**Search results**.

Search results and reasons for study exclusion are outlined in Figure 1. Sixteen studies were excluded from twenty potentially relevant studies. All of the excluded studies in the English language were a case-series design (Level IV). Overall, four studies met the inclusion criteria for this review[8,27-29]. All were published between 2000 and 2010.

### Methodological quality of included studies

Two studies scored ten out of twelve on the Law critical appraisal tool (83%)[27,29]. Two studies (case series) scored nine out of eleven (81%)[8,28]. All four studies provided a description of their sample population, but no study justified its sample size. An adequate description of the process used to calculate sample size is important; inadequate description could potentially indicate sampling bias[30]. Three studies did not adequately control for co-intervention and contamination, making interpretation of their results more difficult[8,27,28]. Two studies included co-morbidities such as meniscal pathology[8,27]. Two studies used magnetic resonance imaging (MRI) to exclude subjects with visible degenerative changes or combined ligament injury[8,28]. Meniscal lesions, if found, were treated concurrently with ACL reconstruction. Individual methodological quality scores are displayed in Table 1.

**Table 1:** Methodological quality

Criteria													
Study	1	2	3	4	5	6	7	8	9	10	11	12	Score
Lavoie et al. [27]	Y	Y	Y	N	Y	Y	N	Y	Y	Y	Y	Y	10/12

Nau et al. [29]	Y	Y	Y	N	Y	Y	Y	Y	Y	N	Y	Y	10/12

Liu et al. [28]	Y	Y	Y	N	Y	Y	N	Y	Y	Y	NA	Y	9/11

Gao et al. [8]	Y	Y	Y	N	Y	Y	N	Y	Y	Y	NA	Y	9/11

NA: not applicable

### Characteristics of studies

One randomised control trial, [29] one cohort study, [27] and two retrospective case series were identified[8,28]. One retrospective case series used a retrospective comparison group[28]. All four studies included populations with chronic ACL ruptures. For the purpose of this review, chronic was defined as being greater than three months from injury to surgery. Follow up periods ranged from 2 months to 5 years. Table 2 provides an overview of the characteristics and post operative rehabilitation protocols.

**Table 2:** Characteristics of included studies

Author (NHMRC level of evidence)	Population (sample size) [Mean age in years]	Comparison	Follow up period (months)	Post operative rehabilitation protocols
Lavoie et al. [27] (III-3)	Chronic & acute (47)[31.6]	**NA**	8-45	**NR**

Nau et al. [29] (II)	Chronic (53)[30.9]	Bone patellar bone autologous graft	2 6 12 24	Identical for both groups WB as tolerated x3/week physiotherapy sessions^#^

Liu et al. [28] (IV)	Chronic (60)[36.0]	**4SHG **autologous graft	48-52	**4SHG Group** Week 0-8: SQ, SLR, Hinged brace Week 1-3: Static step for balance Week 3: Initiated Kn F exercises Week 10: Full WB Week 12:Normal ADL, Kn F > 120° 6 months: RTS (non competitive) 9 months: RTS and all activities **LARS Group** Week 0-1: SQ, SLR, Full Kn F Week 0.5-3: WB with Crutches 2 months: RTS (non competitive) 3-4 months: RTS and all activities

Gao et al. [8] (IV)	Chronic & acute (159)[30.0]	**NA**	36-62	Week 0-1: SQ, Kn F to 90°, crutches, partial WB Week 1-2: Kn F to 120° Week 2-4: progress to full WB 1-2 months: return to full ADLs 3 months: initiate return to jogging 6 months: RTS

**NR: **not reported

**4SHG: **4 strand hamstring graft autologous graft

**NA: **Not applicable

**WB: **weight bearing

**SLR: **Straight leg raise

**SQ: **Static quadriceps exercises

**RTS: **Return to sport

**Kn F**: Knee flexion

**ADLs: **Activities of daily living

*Chronic was termed greater than 3 months from injury time to surgery

**# **For the first 3 months

Two studies used a mixed population of acute and chronic ACL ruptures[8,27]. Lavoie and colleagues included patients with acute or subacute injured knees, in addition to chronic ACL ruptures[27]. No definitions of acute and subacute were provided by the authors. Gao and colleagues included both acute and chronic populations and defined acute injury as duration less than 3 months and chronic as greater than 3 months[8]. The use of mixed populations served to confuse the findings of these studies as it generated a subgroup within their populations. Both studies did not provide a subgroup analysis to compare outcomes in acute and chronic presentations. The cohort study by Lavoie and colleagues included patients with associated pathologies and a history of previous knee surgery, whereas the remaining three studies chose to exclude these patients. Two studies utilised comparison groups; comparing traditional surgical techniques to LARS[28,29].

All four studies differed in their post operative rehabilitation protocols. The first study did not report their protocol[27]. The second study reported using the same protocol for both comparison groups (BPB and LARS), but provided very little detail of the protocol itself[29]. The third study used a similar protocol for both groups with significantly reduced timeframes for their LARS interventional group[28]. The fourth study provided a general outline of their rehabilitation aims for the first 6 months post operatively[8].

### Individual study results

Nau and colleagues compared ipsilateral bone-patellar tendon-bone autologous graft with LARS in a population of chronic, symptomatic, ACL ruptures[29]. This study demonstrated that LARS was comparable to bone patella bone reconstruction in terms of subjective functional scores over a 24 month period. The authors commented on the high likelihood of return to high-level activity in the LARS group, but did not provide statistical analysis to support this contention.

Lavoie and colleagues in a cohort study evaluated patient satisfaction scores for knee stability following ACL reconstructive surgery using LARS[27]. Their study population consisted of subjects with ACL rupture and included associated pathologies such as meniscal tears. This study concluded that the LARS could be considered as a viable option for ACL reconstruction in terms of patient satisfaction. Interestingly, positive patient satisfaction scores were reported despite ongoing knee laxity (average posterior-anterior displacement scores of 7.3 mm).

Liu and colleagues in a retrospective case series compared the effectiveness of the LARS to matched controls who had received traditional ACL reconstruction using a four-strand hamstring autologous graft (4SHG)[28]. All subjects had a period of more than four months since time of injury to time of surgery, and were hence classified as chronic by our definition. This study demonstrated that both the LARS and the 4SHG surgical interventions can result in improvements in functional outcomes after four years.

Gao and colleagues in a retrospective, multicentre case series assessed the clinical outcome of LARS reconstruction, with a 3 to 5 year follow up assessment[8]. LARS surgery was only performed on subjects who, on arthroscopic finding, had a viable ACL stump for the LARS to pass through. Prior to surgery, subjects gave consent for the LARS procedure but were informed that without a viable stump a more traditional approach, either BPB or hamstring tendon autologous graft would be performed. Additional surgical intervention was performed on meniscal injuries, when present. This study concluded that LARS performed in subjects presenting surgically with a viable stump can be a suitable option for ACL reconstruction in terms of function and pain outcomes.

### Outcome measures

A range of subjective and objective outcome measures were used. The International Knee Documentation Committee Subjective Knee Evaluation Form (IKDC) and the Knee injury and Osteoarthritis Outcome Score (KOOS) were used to assess QOL and function. Application of these instruments within each individual study varied considerably. The IKDC assesses symptom, function and sport activity in patients with a variety of knee disorders and has been shown to be a reliable and valid scoring system[31,32]. KOOS, a self administered questionnaire which assesses patient satisfaction, was utilised in two studies[27,29]. The KOOS has been demonstrated to be valid and reliable[33,34].

The Tegner score is an activity grading scale where work and sport level activity is quantified pre-injury, pre-surgery and post-surgery, and was used in all four studies. Both the KT 1000 arthrometer and the Telos Stress System were used to measure structural stability of the knee. The Telos Stress System is a measure of anterior tibial shift relative to the femur, and was used in two studies[27,29]. However, as with other measures, the application of this outcome measure varied between studies. The Lysholm score, which aims to measure change in knee instability, is intended to correspond with the patient's subjective opinion of their function and perceived instability after knee reconstruction. The Lysholm score has questionable psychometric properties[35,36]. Table 3 outlines the outcome measures and outcomes of each study.

**Table 3:** Study outcomes

Study	KOOS	IKDC	Tegner Score	Telos Stress System	KT-1000	Lysholm Scale
Lavoie et al. [27]	NS†	-	S†	NS†	-	-

Nau et al. [29]	S (12 months) NS (24 months)	NS	NS	S^ (6 months) NS (24 months)	-	-

Liu et al. [28]	-	NS	NS	-	S (48 months)	NS

Gao et al. [8]	-	S†^#^	NS†^#^	-	S†	S†

**S: S**ignificant difference

**NS: **No significant difference

**KOOS: **Knee injury and Osteoarthritis Outcome Score

**IKDC: **The International Knee Documentation Committee Subjective Knee Evaluation Form

**BPB: **Bone patellar bone autologous graft

* Significance set at p < 0.05; †Pre-post measures; - outcome measure not used; **# **Final follow up ranged from 36 to 62 months ^ significant difference not in favour of LARS

In addition to these outcomes, one study provided data for isokinetic peak torque testing for quadricep and hamstring muscle groups[8]. In this multicentre study, two out of four clinics had the resources for this type of testing and hence data presented was compiled from 68 of 159 patients (43%). Data were not presented in terms of statistical significance and it was not clear at what stage of follow up data were collected. Post operative knee range of motion and knee stability was assessed using the Lachman and pivot shift tests; manual tests of knee joint stability. Significant differences were reported immediately post operatively for Lachman and pivot shift tests, but long term follow up for these outcomes measures was not reported.

### Complication rates

A number of complications from LARS were reported, including superficial wound infections, graft failure and pain arising from surgical screws. Only one study reported complication related to knee synovitis which may have been secondary to LARS rupture (partial or complete rupture not specified)[8]. Rates of complication for superficial wound infection were 2%, [27] and 1%[8]. Both studies reported that infections resolved with antibiotic treatment.

Device failure, which included either failure of screw fixation or failure of synthetic ligament ranged from 4% to 8%[8,27,29]. Pain relating to surgical screws ranged from less than 1% to 4%[8,27,28]. One study reported three cases of either partial or complete LARS rupture, all linked to sport trauma to the knee at 16, 18 and 21 months post surgery respectively[8]. Tibial or femoral and tibial tunnels were reported to be placed too anteriorly in all three cases, potentially explaining the ongoing instability identified. All three cases underwent revision surgery with traditional techniques (either hamstring allograft or autologous graft) with reported good outcomes.

### Body of Evidence Matrix

The results of the NHMRC body of evidence matrix for this review are presented in Table 4. When reviewing studies within this framework, it is apparent that LARS, as a surgical intervention for symptomatic ACL rupture, should be used with caution. The evidence to date is limited, and as such, cannot support or negate the use of LARS in clinical practice. Further research is required for LARS to be recommended as a suitable, viable and safe option in the management of ACL rupture. It is recommended that due to limitations within the existing evidence base, regular review of progress and evaluation of outcomes should be undertaken as part of implementing the LARS as a surgical intervention.

**Table 4:** Body of Evidence Matrix

Component	Grade	Comments
Evidence Base	**D**-poor Level IV studies, or level I to III studies with high risk of bias	• Four studies • Study design: Randomized Controlled Trial (n = 1), non-randomized, experimental trial (n = 1), case series (n = 2) • Moderate quality of evidence *(refer to quality scores in text)* • 319 participants across four studies. No study justified sample size or performed a power calculation

Consistency	**D**-poor Evidence is inconsistent	• Multiple study designs • Predominantly chronic populations • Differing inclusion criteria in respect to associated pathologies and injury history • Statistical analysis adequate in two out of four studies • Primary outcome measures were abbreviated or modified in two studies^27, 29 ^potentially affecting the reliability and validity of these results.

Clinical Impact	D-poor Slight or restricted	• Effect sizes could not be calculated due to insufficient data reporting • Post operative laxity: Inconsistent findings • Post operative rehabilitation: Protocol adequately described in one study^28^, and omitted in two^27, 29^ • Minimal reporting of outcome measures relating to return to sport • Minimal reporting of objective, functional outcomes • Complication rates were consistently low across all four studies • No follow up greater than 5 years

Generalisability	**C**-satisfactory Population/s studied in body of evidence differ to target population for guideline but it is clinically sensible to apply this evidence to target population	• Higher percentage of male subjects • Age range 18 to 56. One study only provided mean ages of 30.9 and 31 for intervention groups.^29^ • Co-pathologies (previous ACL rupture, associated meniscal or ligamentous injuries), were included in three studies.^8, 28-29^ • All studies used chronic populations. Two study also included acute presentations (< 3 months)^8, 27^ • Mechanism of injury poorly reported • Operative procedure times not reported

Grade of recommendation	**D **Caution Body of evidence is weak and recommendation must be applied with caution	*Current evidence suggests that the use of LARS as a surgical intervention for the treatment of symptomatic ACL deficiency must be considered with caution. Routine use of LARS should be underpinned with regular monitoring of outcomes (subjective and objective) using psychometrically sound instruments. Due to the volume and quality of evidence, current literature on this topic should be interpreted with care*.

## Discussion

This systematic review aimed to evaluate the effectiveness of LARS as a surgical option for symptomatic, anterior cruciate ligament rupture in terms of graft stability, rehabilitation time and return to pre-injury function. This review identified four studies of various designs, of a moderate methodological quality. Graft stability outcomes were found to be inconsistent between the four studies at both post operative and follow up periods. The time frames of rehabilitation periods were poorly reported and at times omitted. Return to pre-injury function and activity was often discussed but not adequately reported in results.

So far, no study has compared LARS and traditional ACL reconstruction methods in terms of return to previous level of function. Furthermore, no study has directly investigated autologous ligament healing along the synthetic meshwork of the LARS, a proposed benefit of LARS over traditional techniques. One study reported finding autologous tissue on the synthetic meshwork in three patients who had undergone revision surgery[8]. The authors of the same study reported that all other patients demonstrated complete autologous tissue covering of the on the synthetic strut. However the method of determining this finding was not reported.

Another proposed benefit of LARS is reduced surgical time[17,18]. Interestingly, no study reported the length of time for LARS ACL reconstruction surgery. A high level of patient satisfaction was the only consistent finding reported in studies.

### Graft stability

It has previously been suggested that the LARS surgical technique may not be appropriate where there is a poor quality ACL stump[17,22]. A viable stump is thought to be important as it allows new ligamentous and neurovascular tissue to regenerate along the synthetic scaffold[17,21]. In chronic cases, scar tissue can interfere with the potential for re-growth[8,17]. Without the support of new ligamentous tissue, the synthetic ligament may be subject to fatigue failure over time[11,14]. All four studies included in this review included patients with chronic ACL ruptures. This could help explain the laxity that was reported in two studies[27,29]. Knee joint laxity may lead to poorer long term outcomes in chronic populations[1,4]. For this reason, LARS ACL reconstruction may be most suited to acute settings where a viable cruciate stump is present.

### Synovitis and graft failure

Previously, high failure rates and a lack of resistance to abrasion lead to a high incidence of reactive synovitis following ACL reconstruction with synthetic grafts[1,11,37]. Only one study included in this review reported a case of knee synovitis. The most common cause of complication was fixation failure, either at the tibial or femoral tunnel, or both. Comparative rates of complications related to fixation failure have been reported for more traditional autologous surgical techniques[1].

### Rehabilitation and return to function

Another proposed benefit of LARS is a reduced risk of graft breakdown in the early phase post-operatively[10]. Due to the potential capacity for early loading it would be inappropriate to stress other types of grafts in the same fashion. Previous literature emphasizes the importance of protecting autologous grafts in the initial stages and has reported high failure rates with early return to impact activity[38,39]. The success of knee reconstruction surgery will therefore not only depend on the surgery, but also the rehabilitation program. Therefore, adequate reporting of rehabilitation programs is essential when determining the effectiveness of ACL reconstruction surgery.

Two studies adequately reported their rehabilitation program[8,28]. The first utilized two different rehabilitation programs for a comparison of 4SHG to the LARS group. The 4SHG group was protected over a period of 3 months and gradually returned to sporting activity over 6-9 months. The LARS group was not protected and non-competitive sports activity was allowed within 2 months with a return to full pre-injury activity between 3-4 months. No differences in terms of functional outcomes were reported. The second study (LARS group only) allowed patients to return to full activities of daily living within 1-2 months, return to jogging at three months and return to sport at 6 months. Both studies allowed weight bearing with crutches, without the use of a knee brace, post operatively.

Nau and colleagues used the same rehabilitation protocol for their LARS and BPB groups[29]. Both groups received physiotherapy input (parameters not specified) three times a week for a total period of three months. This timeframe is not adequate for the BPB grafts as current literature reports ACL rehabilitation for autologous grafts should be a minimum of nine months[39]. One study did not provide any information about their rehabilitation protocol[27]. Although there is a sub-category in the IKDC relating to sporting activity, the IKDC does not specifically assess the timeframe or specific functional requirements of individual sports. Overall no study reported data for timeframes of when patients returned to pre-injury level of function or sport. Therefore recommendations and comparisons in regards to return to pre-injury function or sport and rehabilitation protocols cannot be made based on this review.

### So what/bottom line

As the current body of evidence is limited, the use of LARS to treat symptomatic ACL rupture must be undertaken with caution and respect to individual clinical and organisational circumstances.

To date there is emerging evidence on the benefits associated with LARS surgery, reduced rehabilitative timeframes and early return to pre-injury and/or sports level and therefore it needs to be considered with caution. With regards to complications arising from LARS, the historical finding of increased reactive synovitis was not supported by this review, with only one case reported across all four studies. Furthermore other complications rates were comparable to traditional ACL reconstructive techniques. This is a positive finding which requires ongoing investigations and monitoring.

### Limitations

As with any systematic review, this review has several limitations. There is currently a profound lack of high level, high quality primary evidence to support the use of LARS as a surgical intervention for symptomatic, ACL rupture. The majority of studies were limited in their statistical power by small sample size and sufficient statistical data was often not provided. This limited the amount of comparisons that were able to be made between studies. Furthermore, rehabilitation programs were poorly described and lacked detail. Follow up data were not available beyond 5 years and therefore longer term recommendations cannot be made. The exclusion of studies not published in the English language is a major limitation to this review.

## Conclusions

### Implications for clinical practice

Due to the limited evidence base for LARS for ACL reconstruction, clinical practice continues to be guided by clinician's expertise and experiential knowledge. Low current rupture rate, minimal synovitis, perceived improvements in overall knee stability and possible early return to impact loading activities are some reasons for the use of the synthetic grafts. Synthetic ligaments may be a viable alternative where traditional techniques may not be possible. Examples of this include poor access to allograft, multiple knee surgeries and/or revisions.

### Implications for research

While this systematic review has identified an emerging body of evidence for LARS, it has also recognised important research gaps requiring future prospective investigations. While current research provides positive evidence of patient satisfaction with LARS, it is ambivalent with regards to other measures such as graft stability and long term functional outcomes. Also absent is any research on the cost effectiveness of LARS when compared to other traditional techniques. Therefore, future research should consider important long term outcomes, ideally over a period of five years or longer, which includes outcomes relevant to safety, effectiveness and cost- effectiveness. While the importance of rehabilitation following LARS is well recognised, literature is scant with regards to rehabilitation protocols. This has significant clinical implications as these protocols cannot be replicated in clinical settings due to lack of detail. Therefore, just as the surgical techniques are described in detail, future research should sufficiently describe and implement rehabilitation protocols that are well structured and appropriately designed.

## Declaration of competing interests

WD performs LARS surgery at the Wakefield Orthopaedic Clinic in Adelaide, Australia. He does not receive financial reimbursements, fees, funding or salary from any organisation associated with LARS. All authors declare that they have no competing interests or external financial support.

## Authors' contributions

ZM conceptualized the topic and devised the search strategy and carried out the initial search. ZM, IS and SK assessed inclusion of studies into this review and independently assessed the quality of studies. Data extraction was carried out by ZM and IS. WD provided background literature and clinical expertise for discussion. SK and LP added to discussion and conclusion sections and performed editing roles. All authors have read and approved the manuscript.

## Supplementary Material

Additional file 1 **Modified Critical Review Form**. Click here for file

## References

[B1] George MS Dunn WR Spindler KP Current Concepts Review: Revision Anterior Cruciate Ligament Reconstruction Am J Sports Med 2006 34 12 2026 37 10.1177/0363546506295026 17092921

[B2] Gianotti SM Marshall SW Hume PA Bunt L Incidence of anterior cruciate ligament injury and other knee ligament injuries: A national population-based study J Sci Med Sport 2009 12 622 627 10.1016/j.jsams.2008.07.005 18835221

[B3] Freeman JW Woods WD Laurencin CT Tissue Engineering of the Anterior Cruciate Ligament Using a Braid-Twist Scaffold Design J Biomech 2007 40 9 2029 2036 10.1016/j.jbiomech.2006.09.025 17097666 PMC2034317

[B4] Chaudhari AMW Briant PL Bevill SL Koo S Andriacchi T Knee Kinematics, Cartilage Morphology, and Osteoarthritis after ACL Injury Med & Sci in Sports & Ex 2008 40 2 215 22 10.1249/mss.0b013e31815cbb0e 18202582

[B5] Aït Si Selmi T Fithian D Neyret P The evolution of osteoarthritis in 103 patients with ACL reconstruction at 17 years follow-up The Knee 2006 13 5 353 58 16935515 10.1016/j.knee.2006.02.014

[B6] Andriacchi T Briant PL Bevill SL Koo S Rotational Changes at the Knee after ACL Injury Cause Cartilage Thinning Clinical Orthopaedics & Related Research 2006 442 39 44 10.1097/01.blo.0000197079.26600.09 16394737

[B7] Meuffels DE Favejee MM Vissers MM Heijboer MP Ten year follow-up study comparing conservative versus operative treatment of anterior cruciate ligament ruptures. A matched-pair analysis of high level athletes Br J Sports Med 2009 43 347 351 10.1136/bjsm.2008.049403 18603576

[B8] Gao K Chen S Wang L Zhang W Anterior Cruciate Ligament Reconstruction With LARS Artificial Ligament: A Multicenter Study With 3- to 5-Year Follow-up J Arthroscopic & Related Surg 2010 26 4 515 523 10.1016/j.arthro.2010.02.001 20362832

[B9] Cohen SB Yucha DT Ciccotti MC Goldstein DT Factors Affecting Patient Selection of Graft Type in Anterior Cruciate Ligament Reconstruction J Arthroscopic & Related Surg 2009 25 9 1006 1010 10.1016/j.arthro.2009.02.010 19732639

[B10] Andersson D Samuelsson K Karlsson J Evidence-Based Medicine Series Systematic Review: Treatment of Anterior Cruciate Ligament Injuries with Special Reference to Surgical Technique and Rehabilitation: An Assessment of Randomized Controlled Trials J Arthroscopic & Related Surg 2009 25 6 653 85 10.1016/j.arthro.2009.04.066 19501297

[B11] Freeman JW Woods MD Laurencin CT Tissue Engineering of the Anterior Cruciate Ligament Using a Braid-Twist Scaffold Design J Biomec 2007 40 9 2029 2036 10.1016/j.jbiomech.2006.09.025 PMC2034317 17097666

[B12] Tibor LM Long JL Schilling PL Lilly RJ Clinical Outcomes After Anterior Cruciate Ligament Reconstruction: A Meta-Analysis of Autograft Versus Allograft Tissue Sports Health: A Multidisciplinary Approach 2010 2 1 56 72 10.1177/1941738109347984 PMC3438864 23015924

[B13] Siebold R Buelow JU Bös L Ellermann A Primary ACL reconstruction with fresh-frozen patellar versus Achilles tendon allografts Archives of Orthopaedic and Trauma Surgery 2003 123 4 180 5 12734717 10.1007/s00402-003-0476-1

[B14] Bernadino S ACL prosthesis any promise for the future? Knee surgery, Sports Traumatology, Arthroscopy 2009 10.1007/s00167-009-0982-y 19915821

[B15] Mascarenhas R MacDonald PB Anterior cruciate ligament reconstruction: a look at prosthetics - past, present and possible future Mcgill J Med 2008 11 1 29 37 18523530 PMC2322926

[B16] Trieb K Blahovec H Brand G In vivo and in vitro cellular ingrowth into a new generation of artificial ligaments Eur Surg Res 2004 36 148 151 10.1159/000077256 15178903

[B17] Dericks G Ligament Advanced Reinforcement system anterior cruciate ligament reconstruction Operative Techniques in Sports Medicine 1995 3 3 187 205 10.1016/S1060-1872(95)80009-3

[B18] Teule JG Posterior cruciate ligament reconstruction: the role of synthetic ligaments Riv It Biol Med 2000 20 3 86 88

[B19] Longo UG Lamberti A Maffulli N Denaro V Tendon augmentation grafts: a systematic review British Medical Bulletin 2010 1 24 10.1093/bmb/ldp051 20047971

[B20] Huang JM Qian W Feng S Zi-Min W Cruciate ligament reconstruction using LARS artificial ligament under arthroscopy: 81 cases report Chin Med J 2010 123 2 160 164 20137364

[B21] Corin group Ligament Augmentation and reconstruction system http://www.larsligament.com.au/Accessed Feb 2010

[B22] Laboreau JP Cazenave A Acute ruptures of the anterior cruciate ligament. Reconstruction by suture on a synthetic reinforcement. Results after five years experience Revue de Chirurgie Orthopédique 1991 7 92 102 1829253

[B23] Merlin T Weston A Tooher R Extending an evidence hierarchy to include topics other than treatment: revising the Australian 'levels of evidence.' BMC Medical Research Methodology 2009 9 34 10.1186/1471-2288-9-34 19519887 PMC2700132

[B24] Law M Stewart D Pollock N Critical review form - Quantitative studies McMaster University: Occupational Therapy Evidence-Based Practice Research Group 1998

[B25] Australian Government NHMRC additional levels of evidence and grades for recommendations for developers of guidelines: stage 2 consultation http://www.nhmrc.gov.au/_files_nhmrc/file/guidelines/Stage%202%20Consultation%20Levels%20and%20Grades.pdf

[B26] Machotka Z Kumar S Perraton LG A systematic review of the literature on the effectiveness of exercise therapy for groin pain in athletes Sports Med Arthroscop, Rehab Therapy & Tech 2009 1 5 10.1186/1758-2555-1-5 PMC2674034 19331695

[B27] Lavoie P Fletcher J Duval N Patient satisfaction needs as related to knee stability and objective findings after ACL reconstruction using the LARS artificial ligament The knee 7 2000 157 163 10.1016/S0968-0160(00)00039-9 10927209

[B28] Liu ZT Zhang XL Jiang Y Zeng BF Four-strand hamstring tendon autograft versus LARS artificial ligament for anterior cruciate ligament reconstruction International Orthopaedics (SICOT) 2010 34 45 49 10.1007/s00264-009-0768-3 PMC2899266 19396441

[B29] Nau T Lavoie P Duval N A new generation of artificial ligaments in reconstruction of the anterior cruciate ligament. A two year follow up of a randomised trial J Bone & Jt Surg Br 2002 84B 356 60 10.1302/0301-620x.84b3.12400 12002492

[B30] Berk RA An Introduction to Sample Selection Bias in Sociological Data American Sociological Review 1983 48 3 386 398 10.2307/2095230

[B31] Anderson AF Irrgang JJ Kocher MS Mann BJ The International Knee Documentation Committee Subjective Knee Evaluation Form Am J Sports Med 2006 34 128 10.1177/0363546505280214 16219941

[B32] Irrgang JJ Anderson AF Boland AL Harner CD Responsiveness of the International Knee Documentation Committee Subjective Knee Form Am J Sports Med 2006 34 1567 73 10.1177/0363546506288855 16870824

[B33] Roos EM Roos HP Lohmander LS Ekdahl C Knee Injury and Osteoarthritis Outcome Score (KOOS): development of a self-administered outcome measure J Orthop Sports Phys Therapy 1998 28 2 88 96 10.2519/jospt.1998.28.2.88 9699158

[B34] Roos EW Lohmander LS The Knee injury and Osteoarthritis Outcome Score (KOOS): from joint injury to osteoarthritis Health Qual Life Outcomes 2003 1 64 10.1186/1477-7525-1-64 14613558 PMC280702

[B35] Briggs KK Lysholm J Tegner Y Rodkey WG The Reliability, Validity, and Responsiveness of the Lysholm Score and Tegner Activity Scale for Anterior Cruciate Ligament Injuries of the Knee Am J Sports Med 2009 37 890 897 10.1177/0363546508330143 19261899

[B36] Bengtsson J Möllborg J Werner S A study for testing the sensitivity and reliability of the Lysholm knee scoring scale Knee Surgery, Sports Traumatology, Arthroscopy 1996 4 1 27 31 10.1007/BF01565994 8819060

[B37] Vergis A Gillquist J Current concepts: graft failure in intra-articular anterior cruciate ligament reconstructions: A review of the literature The Journal of Arthroscopic & Related Surgery 1995 11 3 312 321 10.1016/0749-8063(95)90009-8 7632308

[B38] Forster MC Forster IW Patellar tendon or four-strand hamstring? A systematic review of autografts for anterior cruciate ligament reconstruction The Knee 2004 12 3 225 30 10.1007/s00167-003-0475-3 15911297

[B39] Myklebust G Bahr R Return to play guidelines after anterior cruciate ligament surgery Br J Sports Med 2005 39 127 131 10.1136/bjsm.2004.010900 15728687 PMC1725142

