# Supplementary material for: Anterior cruciate ligament repair with LARS (ligament advanced reinforcement system): a systematic review
Source: Sports Med Arthrosc Rehabil Ther Technol. 2010 Dec 7;2:29. doi: 10.1186/1758-2555-2-29 (PMC3016369; doi:10.1186/1758-2555-2-29)
Supplement: Additional file 1 — Modified Critical Review Form. [file 1758-2555-2-29-S1.DOC]

# Appendix

**Appendix 1:** Modified Critical review form – Quantitative studies

CITATION:

Law M, Stewart D, Pollock N, *et al.*: **Critical review form – Quantitative studies.** *McMaster University: Occupational Therapy Evidence-Based Practice Research Group* 1998.

| **STUDY PURPOSE(1)**  Was the purpose stated clearly?  *YES*  *NO* | Outline the purpose of the study. How does the study apply to OT and/or your research question? |
| --- | --- |
| **LITERATURE(1)**  Was relevant background literature reviewed?  *YES*  *NO* | Describe the justification of the need for this study |
| **SAMPLE (2)**  Was the sample described in detail?  *YES*  *NO*  Was the sample size justified?  *YES*  *NO*  *N/A* | Sampling (who; characteristics; how many; how was sampling done?)  IF more than one group, was there similarity between the groups?  N=  Describe ethics procedure. Was informed consent obtained? |
| **OUTCOMES (1)**  Were the outcomes measures reliable & valid?  *YES NO Not addressed* | Specify the frequency of outcome measures (pre, post, follow-up)  Outcome areas(eg: self-care, productivity, leisure)  List measures used |
| **INTERVENTION (2)**  Intervention was described in detail?  *YES NO*  *Not addressed*  Contamination & co-intervention was avoided?  *YES*  *NO*  *Not addressed*  *N/A* | Provide a short description of the intervention (focus, who delivered it, how often, setting). Could the intervention be replicated in practice? |
| **RESULTS (4)**  Results were reported in terms of statistical significance?  *YES*  *NO*  *Not addressed*  *N/A*  Were the analysis method(s) appropriate?  *YES*  *NO*  *Not addressed*  Clinical importance was reported?  *YES*  *NO*  *Not addressed*  Drop outs were reported?  *YES*  *NO* | What were the results? Were they statistically significant (ie p<0.05)? If not statistically significant, was study big enough to show an important difference if it should occur? If there were multiple outcomes, was that take into account for the statistical analysis?  What was the clinical importance of the results? Were the differences between groups clinically meaningful?  Did any participants drop out from the study? Why? (were reasons given and were drop-outs handled appropriately?) |
| **CONCLUSIONS AND CLINICAL IMPLICATIONS (1)**  Conclusions were appropriate given study methods and results  *YES*  *NO* | What did the study conclude? What are the implications of these results for occupational therapy practice? What were the main limitations or biases in the study? |
